# Costs of productivity loss due to occupational cancer in Canada: estimation using claims data from Workers’ Compensation Boards

**DOI:** 10.1186/s13561-017-0145-7

**Published:** 2017-02-10

**Authors:** W. Dominika Wranik, Adam Muir, Min Hu

**Affiliations:** 1 School of Public Administration, Department of Community Health and Epidemiology, Dalhousie University, Halifax, NS Canada; 2 Department of Community Health and Epidemiology, Dalhousie University, Halifax, NS Canada; 3 Department of Economics, Dalhousie University, Halifax, NS Canada

## Abstract

**Introduction:**

Cancer is a leading cause of illness globally, yet our understanding of the financial implications of cancer caused by working conditions and environments is limited. The goal of this study is to estimate the costs of productivity losses due to occupational cancer in Canada, and to evaluate the factors associated with these costs.

**Methods:**

Two sources of data are used: (i) Individual level administrative claims data from the Workers Compensation Board of Nova Scotia; and (ii) provincial aggregated cancer claims statistics from the Association of Workers Compensation Boards of Canada. Benefits paid to claimants are based on actuarial estimates of wage-loss, but do not include medical costs that are covered by the Canadian publicly funded healthcare system. Regional claims level data are used to estimate the total and average (per claim) cost of occupational cancer to the insurance system, and to assess which characteristics of the claim/claimant influence costs. Cost estimates from one region are weighted using regional multipliers to adjust for system differences between regions, and extrapolated to estimate national costs of occupational cancer.

**Results/Discussion:**

We estimate that the total cost of occupational cancer to the Workers’ Compensation system in Canada between 1996 and 2013 was $1.2 billion. The average annual cost was $68 million. The cancer being identified as asbestos related were significantly positively associated with costs, whereas the age of the claimant was significantly negatively associated with costs. The industry type/region, injury type or part of body affected by cancer were not significant cost determinants.

**Conclusion:**

Given the severity of the cancer burden, it is important to understand the financial implications of the disease on workers. Our study shows that productivity losses associated with cancer in the workplace are not negligible, particularly for workers exposed to asbestos.

**Electronic supplementary material:**

The online version of this article (doi:10.1186/s13561-017-0145-7) contains supplementary material, which is available to authorized users.

## Background

The incidence of cancer in Canada was higher than the global average in 2012; more than 290 individuals per 100,000 population were diagnosed with cancer, as compared to a global average of approximately 190 per 100,000 [1] This can create an emotional and financial burden on patients and their families, the latter in the form of health care costs, and also costs of missed employment.

Health care costs to individuals are defrayed in Canada by virtue of the health care system being predominantly publicly funded from general taxation revenues. While drugs are typically not included on the public reimbursement list, many cancer drugs are funded publically [2]. Costs of lost earnings can also be partially defrayed for workers whose cancer diagnosis can be attributed to their working conditions or environment. In those cases, workers can lay claims against their employer.

The Workers’ Compensation system in Canada is an insurance system that protects employers against the risk of work-related injury claims. It was established in the early parts of the 20^th^ century. The general premise behind the program is that workers relinquish their right to sue employers in the event of workplace injury, but gain compensation benefits in exchange [3]. Each injury/fatality claim is carefully reviewed to establish attribution of the injury or illness to workplace conditions. The Workers’ Compensation system in Canada has been characterized as parallel to the publicly funded provincial insurance [3].

When workers in Canada develop cancer that is attributable to the conditions or environments of their workplace, typically referred to as occupational cancer [4], they may file a claim with the Workers’ Compensation Board (WCB). Even though several types of industry have been identified as posing a higher risk of cancer for their workers (construction, fire-fighting, mining, etc.) [5], the specific causes of any individual’s cancer can be challenging to identify, however. Multiple factors can contribute to the illness, and there is often latency between cause and diagnosis [6, 7]. Claims may therefore be rejected.

Canada has 12 WCBs, individually representing each of the provinces and territories, with the exception of Northwest Territories and Nunavut, which share a program. Table 1 outlines the characteristics of these provincial boards. The Workers’ Compensation system has been developing and evolving over the majority of the previous century. Not unlike other national systems in Canada, it has evolved at different speeds and in different directions in the various jurisdictions. The status quo is such that the features of the WCB vary across provinces in the amounts that a worker can expect to receive in compensation, the percentage of regular earnings recovered, and the requirements placed on employers in the case of a workplace injury.

**Table 1:** Characteristics of Workers’ Compensation Boards in Canada

Province	Name of board	Year	Max. compensated earnings	% of earnings (basis for benefits)
Alberta	Workers’ Compensation Board of Alberta	1918	$95,300	90% net
British Columbia	WorkSafeBC	1917	$78,600	90% net
Manitoba	Workers’ Compensation Board of Manitoba	1917	$121,000	90% net
New Brunswick	WorkSafeNB	1919	$60,900	85% loss of earnings
Newfoundland and Labrador	Workplace Health, Safety and Compensation Commission	1951	$61,615	90% net
Northwest Territories/Nunavut	Workers’ Safety & Compensation Commission	1977	$86,000	90% net
Nova Scotia	Workers’ Compensation Board of Nova Scotia	1915	$56,800	75% net (26 weeks) then 85% net
Ontario	Workplace Safety and Insurance Board	1915	$85,200	85% net
Prince Edward Island	Workers’ Compensation Board of Prince Edward Island	1949	$52,100	80% net (38 weeks) then 85% net
Quebec	Commission de la santé et de la sécurité du travail	1931	$70,000	90% net
Saskatchewan	Workers Compensation Board of Saskatchewan	1929	$65,130	90% net
Yukon	Yukon Workers’ Compensation Health & Safety Board	1973	$77,610	75% gross

The amounts of benefits paid to workers by a Workers’ Compensation Board are based on an actuarial estimation of earnings losses that occur as a result of the injury or illness. As such, the amount of benefits can serve as a proxy to understanding the amount of wage loss, which in turn signals productivity loss resulting from a specific injury or illness.

## Literature

Occupational cancer is the leading cause of work-related death in Canada and rates of accepted claims have generally increased since 1997 in Canada [8, 9] and the United Kingdom [10]. Moreover, asbestos-related cancer accounted for nearly 70% of all compensated deaths and most typically affect those with manual labour professions [8, 11]. While the incidence of these reports are clear, measuring the cost of occupational cancer remains difficult.

Little is known about the costs of occupational cancer to a health care system, or any of its components. Estimates in the literature rely on administrative records, national aggregate statistics, and/or questionnaires to estimate occupational cancer costs. All rely on assumptions made about the transferability of incomplete or imperfect data to estimate the incidence and/or prevalence of occupational cancer and/or its cost. For this reason, there is a limited number of published studies that estimate the burden and/or costs of occupational cancer (Additional file 1: Appendix A1).

The creativity of some approaches published in the literature signals the difficulty of finding reliable data regarding the costs of occupational cancer. For example, Fritschi and Driscoll [12] use Finnish estimates of the proportion of cancers caused by occupation to estimate occupational cancer rates in Australia. They use EU estimates of the proportion of workers exposed to carcinogens and apply to Australian industry profiles [12]. Other studies of occupational cancer do not contain cost estimates. Despite limited academic study, especially in Canada, some conclusions can be drawn regarding the nature of occupational cancer and its labour impact, and provide the basis for exploring new methods to estimate costs.

Internationally, the impact of occupational cancer is significant when measured in terms of mortality. Two studies use national mortality data to estimate the number of potential or expected years of life and/or working life lost due to occupational cancer. Binazzi et al. (2013) estimate that on aggregate 170,000 potential years of life and 16,000 potential years of working life were lost due to occupational cancer in Italy in 2006 [13]. Lee et al. (2012) estimate that in Taiwan, between 1997 and 2005, the expected years of life lost per individual were between 5 and 18 on average, depending on the type of cancer [14].

The financial cost of occupational cancer to health systems internationally is also extensive. Estimations of the monetized costs of cancer vary across regions, years, and the specific types of costs included in the calculation. For example, work attributable cancers are estimated to have cost the Spanish Basque health system close to €10 million in 2008 [15]. Costs for all of France in 2010 are estimated between €917 million and €2.18 billion, including direct and indirect social costs [16]. In contrast, O’Neill estimates the cost of work-related cancers in the UK to be in the order of £30 to £60 billion per year, which is a much higher estimate [17].

The cost of occupational cancer in Canada is comparable to international estimates, but the Canadian literature employs a multitude of measurement strategies, particularly at the provincial levels. For example, Hopkins et al. [18] use data from the Canadian Community Health Survey, as well as published numbers from the literature to estimate the national-level cost of occupational cancer in terms of wage loss in 2009. They estimate that workers (patients) and their families have lost $ 3.18 billion [18]. Orenstein et al. [19] estimate that the indirect costs (loss of economic resources and reduced productivity) in Alberta alone are approximately $64 million per year, and that the province incurs approximately $16 million per year in medical system costs. While Quebec estimates that occupational diseases account for approximately $834 million dollars annually in worker’s compensation claims and occupational disease related deaths cost approximately $128 million, exact figures regarding the cost of occupational cancer were unclear [11]. Additionally, the number of compensated occupational cancer claims has also grown progressively in Ontario, however the true burden of occupational cancer is yet to be properly estimated [8]. Due to the lack of literature focusing on all Canadian provinces, particularly Nova Scotia, understanding the cost of occupational cancer is relatively unknown. Estimating and exploring the determinants of the cost of occupational cancer claims in Nova Scotia, as well as nationally by province must be attempted.

## Methods

The goal of this study is twofold: (i) to understand the structure of occupational cancer costs borne by the WCB in Nova Scotia, and (ii) to estimate the national burden of occupational cancer using the NS data.

Two models are developed, a regional model and a national model. The regional model estimates the total costs and average costs per cancer related claim, and the determinants of costs at the level of the province (Nova Scotia). The national model extrapolates national level costs from the regional level using NS average cost per claim, the number of claims per province/territory per year, and a weighing technique to account for differences in the provincial/territorial WCB systems.

### Data

We use two sources of data: (i) the Nova Scotia Workers’ Compensation Board (WCB) administrative claims records, and (ii) the Association of Workers’ Compensation Boards of Canada (AWCBC) aggregated statistics available online or through customized order.

The Nova Scotia WCB records were made available at the individual claims level from 1957 to 2015 and includes all claims with and without time-loss. The records include the short and long term earnings loss benefits paid to individuals up until September 22^nd^, 2015. Other variables available were age in years at the time of the biopsy (<50, 51–64, 65+), industry that the incident occurred (government, construction, manufacturing, and other), type of cancer (occupational, asbestos, fire fighter, and missing), type of injury (Asbestosis, Leukemias, Lymphosarcoma and Reticulosarcoma, neoplasms and tumors, Mesothelioma, other, and unknown), region injury occurred (Halifax-East Shore-West Hants, Annapolis Valley-South Shore-South West, Colchester-East Hants-Cumberland-Pictou, Cape Breton-Guysborough-Antigonish, other, and missing), and type of body part affected (abdomen/digestive, urinary systems, body systems, respiratory system, circulatory system, head and neck, pelvic region, other, and missing). The categorization within variables was exploratory and largely dictated by the nature of the WCB records. Where appropriate categories within variables were collapsed. There were 385 occupational related cancer claims accepted by the Nova Scotia WCB. Claims were dropped from analysis if there was no cost accrued or reported by the WCB (21.0%). Overall, 304 claims with 298 men and six women were included in this study.

For the national model, we used two data-sets from the AWCBC:

1. The total annual costs of all claims and the number of time-loss claims per province/territory for the years 1996 to 2013 was obtained through the online request (http://awcbc.org/?page_id=14). Cost per claim per province per year was calculated (not cancer specific).
2. The number of time-loss claims per province/territory per year for the years 1996 to 2013 for each injury/fatality type, including cancer was obtained through customized order. The full list of cancer types included is in Additional file 1: Appendix A2.

Disaggregated claims-level data are not available through the AWCBC.

### Analysis

#### Regional model

Total cancer cost in Nova Scotia (*TC*_*NS*_) included individual short term disability benefits, long term disability benefits, and medical costs. The total cost per claim was calculated as the summation of annual costs per claim discounted by inflation.

1 $$ T{C}_{NS} = {\displaystyle \sum } T{C}_{NS, t}^{pc}*{\pi}_t $$

Where *TC*_*NS*_ is the total cost in Nova Scotia, $T{C}_{NS, t}^{pc}$ is the total cost per claim in Nova Scotia in year t, and *π*_*t*_ is inflation in year t.

To account for inflation, we used the Consumer Price Index (CPI) base year 2014 data from Statistics Canada [20]. Assumptions about the region and composition of the CPI were required. Furthermore, assumptions about the year(s) of payout for each claimant were required, as this was missing from the data. As a result, we provide eight estimates of *TC*_*NS*_ (Table 2) for comparison of the implications of assumptions.

**Table 2:** Combinations of assumptions used for inflation adjustment of costs

	CPI – national level		CPI – national level	
	CPI – all items	CPI – health and personal care	CPI – all items	CPI – Health and personal care
Year (First Year of Claim)	Cost Estimate 1	Cost Estimate 2	Cost Estimate 5	Cost Estimate 6
Year (Median Year of Claim)	Cost Estimate 3	Cost Estimate 4	Cost Estimate 7	Cost Estimate 8

First, the CPI is available at the national level, and since 1979 it is also at the provincial/territorial levels. Cost calculations using provincial CPI values are therefore challenging for years prior to 1979, and the national CPI is used in those years. This is compared to cost calculations using the national CPI for all years 1957 to 2015. Second, the CPI is available for all goods and services, and it is also available specifically for goods and services related specifically to health and personal care. Estimates using both are compared. Third, for purposes of inflation adjustment, assumptions had to be made about the year in which benefits were paid to claimants. Dates of payments were not available from WCB, and dates when claims were closed were deemed unreliable, because claims were often re-opened. We assumed that short term disability benefits were paid in full in the year the claim was filed and inflation adjustment was done in that year. Long term disability benefits are paid out over a number of years after the claim is filed, however. Two different years of payout were assumed for purposes of inflation adjustment: the first year the claim was filed, and the median year between the first and last years that the claim was open.

#### Regional model – determinants of total costs per cancer-related claim

The determinants of total costs per claim were assessed by estimating the associations between total costs and claim characteristics. Total cost $\left( T{C}_{NS}^{pc}\right)$ did not have a normal distribution and required a natural log transformation to satisfy assumptions necessary to perform linear regression. Univariate analyses, full-model multiple linear regression, and a parsimonious- multiple regression model on natural log transformed total cost were conducted. Equation [2] shows the approach used to estimate the drivers of inflation adjusted total cost per cancer claim.

2 $$ L n\left( T{C}_{NS}^{pc}\right)=\alpha + \boldsymbol{X}\boldsymbol{\hbox{'}}\boldsymbol{\beta} + \varepsilon $$

Where *α* is the intercept, ***X***’ are the claims characteristics (injury type, cancer type, body part affected, age of claimant at biopsy, industry type, region), ***β*** is a vector of estimated coefficients, and *ε* is the error term.

#### National model

The WCB benefits costs related to occupational cancer in Canada were estimated in a series of three steps: (1) regional model estimation of average cost per claim (NS WCB data); (2) estimation of provincial multipliers to capture the relative differences between Provinces (AWCBC data); and (3) estimation of annual and total costs of occupational cancer in Canada by province/territory (NS WCB and AWCBC data).

The average cost per claim in Nova Scotia was calculated using the estimates from the regional model. Equation [1] shows the approach used to estimate the average cost (AC) per claim in Nova Scotia.

3 $$ A{C}_{NS}^{pc}=\frac{T{C}_{NS}}{n_{NS}} $$

Where $A{C}_{NS}^{pc}$ is the average cost per claim, *TC*_*NS*_ is the total cost per claim, and *n*_*NS*_ is the number of claims in Nova Scotia. The confidence interval for the $A{C}_{NS}^{pc}$ is found as follows:

$$ 95\%\  C{I}_{NS}= A{C}_{NS}^{pc}\pm 1.96*\frac{\sigma_{NS}}{\sqrt{n_{NS}}} $$

Provincial multipliers introduced here are weighted indices developed to account for general differences in the WSB systems across provinces, specifically for the systemic relative differences in the costs of claims. Systemic relative differences refer to those outlined in Table 1, namely differences in the maximum compensated earnings, and the percentages of earnings considered as a basis for benefits. The multipliers are calculated using the average cost per claim in each province for all types of claims, not restricted to cancer, including short term and long term benefit costs, but not administrative costs (using AWCBC data).^1^ For each province, we calculate an annual average cost per claim $AA{C}_{it}^{pc}$, where *i* is the province and *t* is the year. The multiplier reflects the relative size of the average cost per claim in province *i* in relation to Nova Scotia in each year (Eq. 4).

4 $$ AA{C}_{it}^{pc}=\frac{T{C}_{it}}{n_{it}} $$

The multiplier is calculated as per equation [5], where we have designated Nova Scotia as the numeraire province:

5 $$ {M}_{it}=\frac{AA{ C}_{it}^{pc}}{AA{ C}_{NS, t}^{pc}} $$

The approach that was used to estimate the average cost of time-loss claims related to occupational cancer from the perspective of the WCB per claim per province is shown in equations [6] and [7]. We assume that all claims in Canada are independent and identically distributed, and follow the same distribution as claims in Nova Scotia, with the same mean and standard deviation. To derive mean and standard deviation for province *i*, we adjust for the mean provincial multiplier.

6 $$ A{C}_i = \overline{M_{i t}}*\  A{C}_{NS} $$

7 $$ {\sigma}_i = \overline{M_{i t}}*\ {\sigma}_{NS} $$

This is the average cost per claim in Nova Scotia discounted by the provincial multiplier. The average cost per claim, standard deviation, and 95% confidence intervals for Canada as a whole are found as per equations [8], [9] and [10].

8 $$ A{C}_{canada}=\frac{{\displaystyle {\sum}_i}\left( A{C}_i*{n}_i\right)}{{\displaystyle {\sum}_i}{n}_i} $$

9 $$ {\sigma}_{canada}=\sqrt{{\displaystyle {\sum}_i}{\left(\frac{n_i}{{\displaystyle {\sum}_i}{n}_i}*{\sigma}_i\right)}^2} $$

10 $$ 95\%\  C{I}_i= A{C}_i\pm {t}_{0.95,{n}_i}*\frac{\sigma_i}{\sqrt{n_i}} $$

This approach to the calculation of national level costs is unique and to the best of our knowledge, has not been used in the literature.

## Results

### Regional model

Descriptive statistics are reported for the full set of Nova Scotia WCB administrative claims related to occupational cancer. Table 3 shows the characteristics of the 304 records from 1957 to 2015. The majority of claims (88.16%) were made at a biopsy age of over 50 years, approximately half (50.99%) were from the manufacturing sector, and claims typically came from the Nova Scotia regions of Cape Breton, Guysborough, and Antigonish. Government workers (32.24%) made a higher percentage of claims than workers in construction (6.25%) and other industries (10.53%). The public sector in Nova Scotia employs many occupations, including construction, therefore the distinction may be blurred. The most common type of occupational cancer is unspecified (49.01%), most often affects the respiratory system (55.92%) and the cancer usually manifests as neoplasms and tumours (50.66%). Asbestos exposure was the most common (26.97%) form of unspecified cancer claim.

**Table 3:** Characteristics of Nova Scotia Workers’ Compensation Board administrative cancer claims records from 1957–2015 (*N* = 304)

	Proportion (%)
Age at biopsy	
<=50	11.84
51–64	44.41
65+	43.75
Industry	
Government	32.24
Construction	6.25
Manufacturing	50.99
Other	10.53
Cancer type	
Occupational	49.01
Asbestos	26.97
Fire Fighter	22.70
Missing	1.32
Injury type	
Asbestosis	8.55
Leukemias	2.63
Lymphosarcoma and Reticulosarcoma	2.30
Neoplasms and Tumors	50.66
Mesothelioma	11.84
Other	14.47
Unknown	9.54
Region	
Halifax, East shore, West Hants	17.43
Annapolis Valley, South Shore, South West	1.97
Colchester-East Hants, Cumberland, Pictou	3.62
Cape Breton, Guysborough, Antigonish	35.86
Other	0.66
Missing	40.46
Body part affected	
Abdomen/Digestive	10.86
Urinary System	3.62
Body Systems	3.29
Respiratory System	55.92
Circulatory System	5.26
Circulatory SystemHead and Neck	4.93
Pelvic Region	2.96
Other	3.95
Missing	9.21

*n* = 304

Estimates of the total costs (*TC*_*NS*_) and average cost per claim $$ \left( A{C}_{NS}^{pc}\right) $$ of occupational cancer in Nova Scotia are presented in Table 4. Eight estimates are presented according to the assumptions made about inflation, as discussed above (figure 1). The range of total cost estimates was between $36.5 million (CPI regional, health and personal care, last year) and $44.0 million (CPI regional, all items, median year). The range of average cost per claim was between $120,182 and $145,807. Assumptions about the CPI influenced the estimates, but differences in estimates were not statistically significant. It is important to note that the cost estimates may change over time, because several claims are still open and continue to accrue costs.

**Table 4:** Nova Scotia - total cost of all cancer claims and the average cost of cancer claims (95% confidence intervals, adjusted for inflation using combinations of three assumptions: CPI region; CPI composition; year of claim)

Cost estimate	CPI assumptions (2014 base year)			Total cost ($ ‘000)	Average cost ($ ‘000)	95% CI ($ ‘000)
1	National	Last Year	All Items	37 500	123	107 – 140
2			Health^b^	36 800	121	105 – 137
3		Median Year	All Items	43 100	142	122 – 162
4			Health^b^	41 400	136	117 - 155
5	Regional^a^	Last Year	All Items	37 900	125	108 – 141
6			Health^b^	36 500	120	104 – 136
7		Median Year	All Items	44 000	145	124 – 165
8			Health^b^	40 500	133	115 – 152

^a^Combination of the National CPI until 1979 and the Nova Scotia CPI in 1979 and thereafter

^b^Health and Personal Care Items

The analysis of the determinants of the cost per claim in Nova Scotia presented here focuses on cost estimate 8, based on the regional health related CPI and using the midpoint year. Results do not appear to be sensitive to the choice of cost estimate (Additional file 1: Appendix A3). Results of unadjusted (univariate) and adjusted (multivariate) linear regression models of natural log transformed cost estimates are provided in Table 5 for both a full and a parsimonious model. The full-model included, age in years at the time of the biopsy, industry that the incident occurred, type of cancer, type of injury, region, and type of body part affected. The parsimonious model includes age at the time of the biopsy, industry that the incident occurred, and cancer type. All models indicate p-values including, *p* < 0.01, *p* < 0.05, and *p* < 0.1. All beta-coefficients are exponentiated and expressed as a percentage of the effect on total cost compared to the referent.

**Table 5:** Determinants of occupational cancer costs in Nova Scotia – cost estimate 8

	Unadjusted	Adjusted full model	Adjusted parsimonious model
Age at biopsy			
<=50	1	1	1
51–64	4.13%	−21.69%	−21.11%
65+	−67.08%*	−81.83%***	−79.81%***
Industry			
Government	1	1	1
Construction	173.74%	173.22%	163.21%
Manufacturing	−0.32%	6.30%	55.19%
Other	−71.96%*	−61.38%	−64.75%
Cancer type			
Occupational	1	1	1
Asbestos	362.61%***	1308.63%***	555.68%***
Fire Fighter	94.57%	165.25%	140.73%
Missing	513.98%	619.51%	759.43%
Injury type			
Asbestosis	1	1	
Leukemias	158.78%	377.98%	
Lymphosarcoma and Reticulosarcoma	−30.25%	96.03%	
Neoplasms and Tumors	5.13%	84.67%	
Mesothelioma	82.63%	13.10%	
Other	201.89%	620.30%**	
Unknown	−46.15%	72.77%	
Region			
Halifax, East shore, West Hants	1	1	
Annapolis Valley, South Shore, South West	114.26%	26.59%	
Colchester-East Hants, Cumberland, Pictou	−59.90%	−2.97%	
Cape Breton, Guysborough, Antigonish	−36.17%	16.88%	
Other	−98.58%*	−98.92%**	
Missing	−73.96%**	−50.63%	
Body part affected			
Abdomen/Digestive	1	1	
Urinary System	126.23%	126.42%	
Body Systems	185.17%	26.36%	
Respiratory System	8.94%	2.99%	
Circulatory System	−31.00%	−52.84%	
Head and Neck	−73.36%	−74.76%	
Pelvic Region	−37.69%	−3.41%	
Other	−38.65%	−69.28%	
Missing	−63.66%	−81.03%	

Unadjusted and adjusted linear regression models were log transformed. Values shown are exponentiated to estimate the geometric mean, expressed as a percentage of change in total cost compared to the referent

Estimates are adjusted for the national (1957–1978) and Nova Scotia (1979–2015) Consumer Price Index for Health and Personal Care

Inflation was determined using year of biopsy

***p < 0.01, **p < 0.05, *P < 0.1

*N* = 304

Total cost: $40 500 000

The bold-face entries are highlighting those results that are statistically significant

Overall, our results suggest that the average costs per WCB cancer claim are influenced by the age of the claimant and the cancer type being related to asbestos.

Specifically, results show that the costs of claims of individuals who were 65 years and older at time of biopsy were significantly lower compared to individuals 50 years or younger. Cost were lower by approximately 67% in the unadjusted model (*p* < 0.1), and 82% in the adjusted full model (*p* < 0.01) and 80% in the parsimonious model (*p* < 0.01). Furthermore, claims for asbestos related cancer were substantively more costly than the general unspecified occupational cancer type. Costs were higher by 363% in the unadjusted model (*p* < 0.01), 1309% higher in the full adjusted model (*p* < 0.01), and 556% higher in the adjusted parsimonious model (*p* < 0.01) relative to unspecified occupational cancer claims. Costs were also influenced by injury type and region within Nova Scotia being reported as ‘other’. The effects of industry type were statistically significant only in the unadjusted model, but became insignificant after adjustment for covariates. Cost did not depend on the body part affected. Cost per claim by gender was not examined because there were too few women in the sample (fewer than 10).

### National model

Results of the estimation of Provincial multipliers are reported in Table 6. Multipliers show the interprovincial variation in the costs of benefits paid by Provincial WCBs across all claims, including but not limited to cancer. A multiplier lower than one indicates that the Province’s WCB typically has lower benefits when compared to Nova Scotia, for example Alberta, British Columbia, Quebec, and Manitoba. A multiplier higher than one indicates that the Province’s WCB typically has higher benefits when compared to Nova Scotia, for example Ontario and New Brunswick.

**Table 6:** Provincial WCB multipliers

Province	Multiplier in 2013	Average multiplier (1996–2013)
Alberta	0.69	0.87
British Columbia	0.78	0.78
Manitoba	0.41	0.39
New Brunswick	0.99	1.22
Newfoundland	0.90	1.09
Nova Scotia	1.00	1.00
Nunavut/NWT	0.87	1.24
Ontario	1.03	1.50
Prince Edward Island	0.85	0.69
Quebec	0.83	0.73
Saskatchewan	0.61	0.54
Yukon	1.24	1.45

The burden of occupational cancer is captured in Table 7 showing the number of claims made in each Province between 1996 and 2013, as well as Canada wide. Nunavut/NWT, New Brunswick and Prince Edward Island had the lowest number of claims, and Ontario, Quebec and British Columbia had the highest number of claims.

**Table 7:** Time-loss claims by province

Province	Number of new claims	Claims in 2013^a^
Alberta	758	83
British Columbia	1242	92
Manitoba	127	12
New Brunswick	17	5
Newfoundland	115	18
Nova Scotia	57	6
Nunavut/NWT	4	-
Ontario	3540	150
Prince Edward Island	10	-
Quebec	2314	175
Saskatchewan	28	-
Yukon	49	-
Total for Canada	8261	541

^a^Provincial reporting is not complete for 2013, some values are missing

The estimated costs of work-related cancer to the WCB system in Canada and by province are shown in Table 8. The average cost of per claim in Nova Scotia is estimated on the basis of the claims-level Nova Scotia data. The average costs per claim for other provinces are estimated using the multiplier approach (based on AWCBC data). Table 8 also reports on the total cost of occupational cancer between 1996 and 2003 for each province, as well as for Canada as a whole. The total cost in Canada between 1996 and 2003 was approximately $1.2 billion, and the average cost per year was approximately $68 million.

**Table 8:** Cost of work-related cancer by province (1996 to 2013)^a^

Province	Total cost ($ ‘000)	95% CI of total cost ($ ‘000)	Average cost per year ($ ‘000)	95% CI of average cost per year ($ ‘000)	Average cost per case ($ ‘000)	95% CI of cost per case ($ ‘000)
Alberta	87,839	(80,138 – 95,539)	4,879	(3,065 – 6,695)	116	(106 – 126)
British Columbia	129,037	(120,199 – 137,874)	7,169	(5,086 – 9,252)	104	(97 – 111)
Manitoba	6,597	(5,184 – 8,010)	439	(75 – 805)	52	(41 – 63)
New Brunswick	2,763	(1,022 – 4,503)	691	(−179 – 1,561)	163	(60 – 265)
Newfoundland	16,696	(12,939 – 20,454)	1,518	(385 – 2,651)	145	(113 – 178)
Nova Scotia	40,492	(38,065 – 42,929)	4,049	(3,282 – 4,817)	133	(91 – 176)
Nunavut/NWT	660	(−469 – 1,789)	661	(−469 – 1,789)	165	(−117 – 447)
Ontario	707,281	(678,589 – 735,973)	39,293	(32,531 – 46,056)	199	(192 – 208)
Prince Edward Island	919	(122 – 1,716)	919	(122 – 1,716)	92	(12 – 172)
Quebec	225,001	(213,711 – 236,289)	12,500	(9,839 – 15,161)	97	(92 – 102)
Saskatchewan	2,014	(1,054 – 2,974)	403	(−26 – 832)	72	(38 – 106)
Yukon	9,464	(6,134 – 12,793)	3,155	(1,232 – 5,077)	193	(125 – 261)
Total for Canada	1,228,763	(1,208,669 – 1,248,856)	68,265	(63,529 – 73,001)	149	(146 – 151)

^a^Rounded to nearest 1000 Canadian dollars, including only years for which data were available. Negative confidence interval caused by small number of observations, and high variance of specific provinces, e.g. NU. There are only 1 year data for both NU and PEI, so their total cost equal average cost per year

## Discussion

Our study explores the determinant of cost of cancer claims in Nova Scotia and provides insight into an area little investigated. Our estimates from the parsimonious model suggest that claims with asbestos related cancer have a fivefold increase in cost compared to unspecified occupational cancer claims. Del Bainco and Demers [8] observed that in Ontario, the number of accepted claims for occupational cancer-related deaths have increased between 1997 and 2010, and that it was most often as a result of exposure to asbestos, commonly experienced in high risk industries. Our results complement their findings, suggesting that while asbestos related cancers are becoming more commonly reported, they are also significantly more costly than other occupational cancer claims. This association is independent of the type of industry in which the claimant acquired the illness. Further investigations into the mechanisms by which asbestos exposure claim increase costs are needed.

We also find that older claimants accrue significantly (80%) lower costs than younger claimants. We have not found comparable findings in the literature. Given that long term benefits primarily reflect lost wages, the likely explanation is that many claimants over the age of 65 do not qualify for wage replacement benefits due to retirement.

Our estimates are conservative estimates of the costs of occupational cancer in Canada as faced by the WCB system. Our results likely underestimate the true costs, because the data available through the AWCBC is not complete, given that it relies on provincial reporting. It is also a conservative estimate, since all claims approved by WCB have been reviewed and determined to be cancers attributed to work conditions. More cases of occupational cancer may exist, but remain unclaimed, or claims are rejected due to insufficient evidence. Discrepancies between the average 1996–2013 costs and the total costs are present, because data are not reported for all years for all provinces. Estimations are based only on reported data. For example, the average cost per year in Manitoba is based on 15 years of data, not 18.

Our estimates of the cancer burden, in terms of number of claims accepted, are relatively lower than those reported in the literature. There are three reasons. First, the AWCBC reports only time-loss claims, and does not include claims of individuals who continue to work while ill. This difference could be substantial. For example, the Nova Scotia dataset records 248 new claims between 1996 and 2013, whereas the AWCBC database records 57 time-loss claims in that same time period, i.e. only 23% of all claims were time-loss claims. Second, the number of claims filed and claims approved by the insurance is naturally lower than the number of cases of occupational cancer, since some patients do not file a claim, and some claims are not approved.

Since 1996, the Canadian WCB system has paid approximately $ 1.2 billion for work related cancer claims, at an average annual cost of approximately $66 million. Ontario faced the highest cost in total and on average, followed by Quebec, British Columbia and Alberta. This is not surprising, given that Ontario has the highest number of approved claims, and pays the highest benefits relative to other provinces. Quebec pays relatively lower benefits, but faces a higher number of approved claims compared to Ontario.

The cost to the WCB insurer does not account for the costs to the health care system that were incurred outside of the WCB claim. Many claimants living with cancer bring their claim to the WCB after the illness has progressed and treatment has begun or has been completed. The WCB does not reimburse the public health system for the costs of care retroactively.

The cost to the WCB insurer serves as a meaningful proxy to the estimation of wage loss due to occupational cancer for workers. It does not account for wage loss due to cancer that is not work-related, nor does it account for the wage loss of family members affected. Furthermore, the payments made by the WCB have upper limits based on the maximum insurable earnings threshold and insure less than 100% of earnings (Table 1). Therefore, our national level estimate of $1.2 billion is lower than the $3 billion estimated by Hopkins et al. [18]. Similarly, our estimate for Alberta is $4.9 million, which is lower than the $64 million estimated by Orenstein et al. [19]. This is consistent with our discussion, since the other studies define productivity and wage loss to include the loss experienced by workers afflicted with cancer directly, and also indirectly through the loss experienced by others in the system, e.g. caregivers. Furthermore, Orenstein et al. rely on an attributable risk approach to estimate the proportion of cancer cases in the province that are liked to working conditions, whereas our study focuses on the number of claims made by workers and accepted by the insurer.

The limitations of our study are twofold. First, we have a relatively small number of the WCB individual claims data from Nova Scotia. Claims due to occupational cancer as a proportion of total WCB claims are less than 1% in most years. Second, the aggregate records available through the AWCBC appear to be incomplete, in particular for the Territories, Saskatchewan and Prince Edward Island, where data is not available for most of the years between 1996 and 2013.

## Conclusion

We find that the Canadian WCB insurance system spends approximately $68 million on occupational cancer claims annually, and has spent approximately $1.2 billion between 1996 and 2013. The study contributes to a very limited body of literature and expands our understanding of the size and determinants of the costs of occupational cancer. The study is based on claims of lost wages laid against employers through the Canadian worker’s compensation insurance system, which serve as an approximation of productivity losses with high face validity.

The need for programs to prevent occupational cancer has long been recognized in Canada [21, 22] and internationally [23]. Yet our data suggest that the number of occupational cancer claims has not been declining over the years, and neither have the costs of claims. Increased funding of for programs to prevent occupational cancer may be a best strategy to cost-savings, not to mention a reduction in the incidence of cancer.

## Additional file

Additional file 1: Appendices. (PDF 587 kb)

## Footnotes

1 The average cost per claim for all claims is likely to be lower than the average cost per claim for cancer claims only. Reliance on all claims in this calculation is likely to introduce bias. Our approach overestimates the costs of cancer for regions with a proportion of cancer cases higher than in Nova Scotia, and vice versa.
