# Supplementary material for: Costs of productivity loss due to occupational cancer in Canada: estimation using claims data from Workers’ Compensation Boards
Source: Health Econ Rev. 2017 Feb 10;7:9. doi: 10.1186/s13561-017-0145-7 (PMC5307412; doi:10.1186/s13561-017-0145-7)
Supplement: Additional file 1: — Appendices. (PDF 587 kb) [file 13561_2017_145_MOESM1_ESM.pdf]

Appendix A1 – Previous studies of occupational cancer burden and/or cost

Authors	Context	Data Sources	Estimated Burden	Estimated Cost
Binazzi et al. (2013)	Italy 2006	Italian National Institute of Statistics – mortality data (occupational cancer deaths	8000-8500 deaths per year from occupational cancer 170,000 potential years of life lost, and 16,000 potential years of working life lost	360,000 Euro in indirect economic costs; 456,000 Euro in health care costs of occupational cancer.
Del Bianco and Demers (2013)	Canada 1997 to 2010	Association of WSBs of Canada, Canadian Cancer Society Cancer Statistics	Deaths from occupational cancer have increased in recent years, accepted claims increased particularly in Ontario	n/a
Fritschi and Driscoll (2006)	Australia	Finnish estimates of proportion of cancers caused by occupation applied to Australian number of cancers. EU estimates of the proportion of workers exposed to carcinogens applied to Australian industrial profiles.	5,000 invasive cancers 34,000 non-melanoma skin cancers caused by carcinogens each year.	n/a
Gomez et al. (2013)	Spain Basque county 2008		1331 work-attributable cancers hospitalizations, 229 specialized ambulatory cases	> 10 million Euro Public health system costs
Hopkins et al., 2010	Canada 2009	Canadian Community Health Survey	Labour participation reduced by 36% ($4518) in cancer patients and workable hours of care givers by 23% ($2887)	Wage loss from cancer $3.18 billion in 2009; Includes loss to patients and caregivers;
Jardine et al. (2015)		Not a peer-reviewed publication, but describes an interesting ongoing research program related to this.		
Labreche et al. (2016)	Quebec, Canada	Finland, UK, work-attributable fractions applied to Canadian Cancer Statistics	6% of cancer incidence attributable to work, avg. 2160 new cancer diagnoses, 1190 cancer deaths per year.	n/a
Lebeau et al. (2014)	Quebec, Canada, 2005 to 2007	Quebec's Workers' Compensation Board (CSST)	$2.84 billon billion to human costs	$4.62 billion on average per year; average cost per case is $38,355 (occupational injuries and diseases)
Lee et al. (2012)	Taiwan 1997-2005	Taiwan Cancer Registry National Mortality Registry	51408 lung, 136 pleural mesothelioma, 12891 bladder, 5285 leukemia, 69720 in total new cases from 1997-2005, followed until 2007; Expected years of life lost estimated	Lifetime healthcare expenditures (mean per person) Us$ 22359 lung Us$ 14900 mesothelioma Us$ 51987 bladder Us$ 59741 leukemia
Leigh et al. (2004)	US, 1999	Workers Compensation Offices from 16 US states	Cost shifting between workers compensation and private health care	$8 to $23 billion in medical costs shifted from WCB to private individuals, insurance or state

Leigh et al. (1997)	U.S, 1992	Bureau of Labour Statistics, National Council on Compensation Insurance, National Centre for Health Statistics, Heath Care Financing Administration data	6500 job-related deaths from injury, 13.2 million nonfatal injuries, 60300 deaths from disease, 862200 illnesses per year	$65 billion in direct costs and $106 billion in indirect cost = $171 billion total costs<br>Injuries - $145 billion<br>Illnesses - $26 billion
O'Neill	UK	Data sources and methods not clear	20000-40000 new cases of work-related cancer each year	Annual cost to the economy of 29.5 to 59 billion pounds
Orenstein et al. 2010	Canada		761 (217 to 786) new occupational cancers in Alberta every year, 2,700 (1520 to 5400) people living with cancer due to occupational exposures.	The direct cost to the medical system approx $15,682,000 per year. Indirect costs—resulting from loss of economic resources and reduced productivity—approx $64.1 million per year.
Rushton et al. 2010	Britain	Literature risk estimates Carcinogen Exposure Database, UK Labour Force Survey, Census of Employment	Estimation of cancer cases attributable to occupation	No cost estimates
Serrier et al. 2014	France 2010	Epidemiological data	Reduction in labour force participation: 36% by patients, and 23% by care-givers.	Social costs between 917 and 2181 million Euro.<br>Including<br>Indirect costs between 119 and 229 million Euro.
Takala et al. (2014)	Global, 2008-2011	ILO, WHO, EU nations	Globally, 2.3 million deaths due to occupational injuries and work related diseases	Estimated 1.6-6.0% of GDP go towards work-related injuries and communicable diseases.

Appendix A2

The following injury/fatality claims were used in the estimations

- Asbestosis, and the following work-related cancers.
- Neoplasms, tumors, and cancer, uns.
- Malignant neoplasms and tumors (cancers, carcinomas, and sarcomas), uns.
- Malignant neoplasms and tumors of bone or connective tissue, uns.
- Bone, articular cartilage
- Connective and other soft tissue
- Malignant neoplasms and tumors of bone or connective tissue n.e.c.
- Malignant neoplasms and tumors of the skin, uns.
- Melanoma of the skin (melanocarcinoma)
- Non-melanoma skin cancer (squamous and basal cell)
- Multiple malignant neoplasms and tumors of the skin
- Malignant neoplasms and tumors of the skin, n.e.c.
- Malignant neoplasms and tumors of lymphatic and hematopoietic tissue, uns.
- Lymphosarcoma and reticulosarcoma (lymphoma, non-hodgkin's lymphoma)
- Hodgkin's disease
- Multiple myeloma
- Leukemias
- Malignant neoplasms and tumors of lymphatic and hematopoietic tissue, n.e.c.
- Malignant neoplasms and tumours of sites, n.e.c.
- Mesothelioma
- Benign neoplasms and tumors, uns.
- Benign neoplasms of bone, connective tissue, and skin, uns.
- Benign neoplasms of bone and articular cartilage
- Lipoma (fatty tumor)
- Other benign neoplasms of connective and other soft tissue
- Benign neoplasms and tumors of other sites, uns.
- Hemangioma and lymphangioma--any site
- Benign neoplasms and tumors of other sites, n.e.c.
- Neoplasms and tumors of unknown properties, uns.

Appendix A3 – Determinants of Occupational Cancer Costs in Nova Scotia, estimates based on various inflation adjustment assumptions

Table A3-1 - Determinants of Occupational Cancer Costs in Nova Scotia – Cost Estimate 1

	Unadjusted	Adjusted Full Model	Adjusted Parsimonious Model
Age at biopsy			
<=50	1	1	1
51-64	39.49%	11.17%	10.27%
65+	-52.79%	**-70.61%****	**-69.87%****
Industry			
Government	**1**	**1**	**1**
Construction	186.48%	161.59%	143.98%
Manufacturing	5.98%	11.68%	37.91%
Other	**-63.33%***	-52.16%	-58.13%
Cancer type			
Occupational	1	1	1
Asbestos	**321.10%*****	**798.54%*****	**463.28%*****
Fire Fighter	59.49%	126.57%	85.11%
Missing	315.12%	433.83%	453.78%
Injury type			
Asbestosis	1	1	
Leukemias	47.81%	89.72%	
Lymphosarcoma and Reticulosarcoma	-59.00%	-25.72%	
Neoplasms and Tumors	-30.95%	8.69%	
Mesothelioma	60.24%	7.94%	
Other	106.78%	**304.31%****	
Unknown	-28.27%	36.40%	
Region			
Halifax, East Shore, West Hants	1	1	
Annapolis Valley, South Shore, South West	78.26%	18.2%	
Colchester-East Hants, Cumberland, Pictou	-65.31%	-21.79%	
Cape Breton, Guysborough, Antigonish	-34.99%	11.71%	
Other	-86.42%	-87.59%	
Missing	**-68.54%*****	-48.86%	
Body part affected			
Abdomen/Digestive	1	1	
Urinary System	94.55%	104.66%	
Body Systems	143.42%	30.08%	
Respiratory System	20.38%	-7.72%	
Circulatory System	-20.08%	-29.49%	
Head and Neck	-68.99%	-70.65%	
Pelvic Region	-47.12%	-30.02%	
Other	-46.45%	-71.00%	
Missing	-25.77%	-65.21%	

Unadjusted and adjusted linear regression models were log transformed. Values shown are exponentiated to estimate the geometric mean, expressed as a percentage of change in total cost compared to the referent
Total cost of claims were adjusted for inflation to 2014 from last year accident claims were reported
Estimates are adjusted for the Consumer Price Index for All items
Inflation was determined using year of biopsy
*** p<0.01, ** p<0.05, * P<0.1
N = 304
Total cost: $37 500 000

Table A3-2 - Determinants of Occupational Cancer Costs in Nova Scotia – Cost Estimate 2

	**Unadjusted**	**Adjusted Full Model**	**Adjusted Parsimonious Model**
Age at biopsy			
<=50	1	1	1
51-64	39.26%	10.85%	10.06%
65+	**-53.20%***	**-71.02%****	**-70.18%*****
Industry			
Government	**1**	**1**	**1**
Construction	186.65%	165.33%	146.75%
Manufacturing	4.75%	12.47%	38.38%
Other	**-63.70%***	-51.90%	-57.97%
Cancer type			
Occupational	1	1	1
Asbestos	**327.42%*****	**808.11%*****	**472.93%*****
Fire Fighter	62.69%	130.94%	89.52%
Missing	317.16%	434.79%	455.83%
Injury type			
Asbestosis	1	1	
Leukemias	49.77%	89.17%	
Lymphosarcoma and Reticulosarcoma	-58.81%	-26.85%	
Neoplasms and Tumors	-31.35%	7.56%	
Mesothelioma	60.95%	7.02%	
Other	102.34%	**298.25%****	
Unknown	-28.89%	34.35%	
Region			
Halifax, East Shore, West Hants	1	1	
Annapolis Valley, South Shore, South West	79.46%	18.01%	
Colchester-East Hants, Cumberland, Pictou	-65.74%	-23.09%	
Cape Breton, Guysborough, Antigonish	-35.95%	10.54%	
Other	-86.60%	-87.66%	
Missing	**-68.39%*****	-48.42%	
Body part affected			
Abdomen/Digestive	1	1	
Urinary System	95.48%	107.84%	
Body Systems	143.22%	29.91%	
Respiratory System	18.41%	-8.32%	
Circulatory System	-19.85%	-29.31%	
Head and Neck	-69.36%	-70.59%	
Pelvic Region	-47.38%	-29.74%	
Other	-46.68%	-71.08%	
Missing	-26.51%	-65.29%	

Unadjusted and adjusted linear regression models were log transformed. Values shown are exponentiated to estimate the geometric mean, expressed as a percentage of change in total cost compared to the referent
Total cost of claims were adjusted for inflation to 2014 from last year accident claims were reported
Estimates are adjusted for the Consumer Price Index for Health and Personal Care.
Inflation was determined using year of biopsy
*** p<0.01, ** p<0.05, * P<0.1
N = 304
Total cost: $36 800 000

Table A3-3 - Determinants of Occupational Cancer Costs in Nova Scotia – Cost Estimate 3

	Unadjusted	Adjusted Full Model	Adjusted Parsimonious Model
Age at biopsy			
<=50	1	1	1
51-64	3.23%	28.76%	-21.87%
65+	**-67.39%***	**-81.85%*****	**-79.94%*****
Industry			
Government	**1**	**1**	**1**
Construction	174.81%	169.64%	160.67%
Manufacturing	1.83%	5.62%	55.27%
Other	**-71.70%***	-61.87%	-65.210%
Cancer type			
Occupational	1	1	1
Asbestos	**348.60%*****	**1295.31%*****	**535.98%*****
Fire Fighter	88.57%	159.37%	132.82%
Missing	524.07%	635.07%	776.79%
Injury type			
Asbestosis	1	1	
Leukemias	156.41%	386.91%	
Lymphosarcoma and Reticulosarcoma	-30.02%	104.62%	
Neoplasms and Tumors	7.62%	90.24%	
Mesothelioma	80.74%	14.37%	
Other	**218.52%***	**652.10%****	
Unknown	-45.27%	78.10%	
Region			
Halifax, East Shore, West Hants	1	1	
Annapolis Valley, South Shore, South West	112.23%	26.58%	
Colchester-East Hants, Cumberland, Pictou	-58.98%	-0.57%	
Cape Breton, Guysborough, Antigonish	-34.45%	18.45%	
Other	**-98.62%***	**-98.97%****	
Missing	**-74.44%*****	-51.56%	
Body part affected			
Abdomen/Digestive	1	1	
Urinary System	127.23%	122.75%	
Body Systems	185.02%	25.83%	
Respiratory System	11.85%	-4.41%	
Circulatory System	-31.49%	-53.36%	
Head and Neck	-72.56%	-74.68%	
Pelvic Region	-37.46%	-3.93%	
Other	-38.36%	-69.34%	
Missing	-63.35%	-81.08%	

Unadjusted and adjusted linear regression models were log transformed. Values shown are exponentiated to estimate the geometric mean, expressed as a percentage of change in total cost compared to the referent

Total cost of claims were adjusted for inflation to 2014 from first year accident claims were reported

Estimates are adjusted for the Consumer Price Index for All items

Inflation was determined using year of biopsy

*** p<0.01, ** p<0.05, * P<0.1

N = 304

Total cost: $43 100 000

Table A3-4 - Determinants of Occupational Cancer Costs in Nova Scotia – Cost Estimate 4

	Unadjusted	Adjusted Full Model	Adjusted Parsimonious Model
Age at biopsy			
<=50	1	1	1
51-64	3.82%	-21.89%	-21.38%
65+	**-67.22%***	**-81.82%*****	**-79.87%*****
Industry			
Government	**1**	**1**	**1**
Construction	175.05%	172.62%	163.16%
Manufacturing	0.60%	6.10%	55.43%
Other	**-71.840%***	-61.54%	-64.88%
Cancer type			
Occupational	1	1	1
Asbestos	**356.86%*****	**1302.72%*****	**547.73%*****
Fire Fighter	92.01%	143.83%	137.71%
Missing	514.91%	398.53%	762.10%
Injury type			
Asbestosis	1	1	
Leukemias	157.54%	348.75%	
Lymphosarcoma and Reticulosarcoma	-30.46%	98.30%	
Neoplasms and Tumors	5.78%	86.06%	
Mesothelioma	81.45%	13.37%	
Other	208.14%	**631.11%****	
Unknown	-45.84%	74.47%	
Region			
Halifax, East Shore, West Hants	1	1	
Annapolis Valley, South Shore, South West	113.93%	26.79%	
Colchester-East Hants, Cumberland, Pictou	-59.54%	-2.04%	
Cape Breton, Guysborough, Antigonish	-35.44%	17.62%	
Other	-98.60%	**-98.94%****	
Missing	**-74.17%*****	-51.07%	
Body part affected			
Abdomen/Digestive	1	1	
Urinary System	126.32%	124.41%	
Body Systems	185.59%	26.19%	
Respiratory System	10.05%	-3.25%	
Circulatory System	-31.23%	-53.09%	
Head and Neck	-73.04%	-74.77%	
Pelvic Region	-37.31%	-3.31%	
Other	-38.44%	-69.28%	
Missing	-63.48%	-81.07%	

Unadjusted and adjusted linear regression models were log transformed. Values shown are exponentiated to estimate the geometric mean, expressed as a percentage of change in total cost compared to the referent
Total cost of claims were adjusted for inflation to 2014 from first year accident claims were reported
Estimates are adjusted for the Consumer Price Index for Health and Personal Care.
Inflation was determined using year of biopsy
*** p<0.01, ** p<0.05, * P<0.1
N = 304
Total cost: $41 400 000

Table A3-5 - Determinants of Occupational Cancer Costs in Nova Scotia – Cost Estimate 5

	Unadjusted	Adjusted Full Model	Adjusted Parsimonious Model
Age at biopsy			
<=50	1	1	1
51-64	39.53%	11.36%	10.33%
65+	-52.73%	**-70.47%****	**-69.80%****
Industry			
Government	**1**	**1**	**1**
Construction	187.00%	160.70%	143.22%
Manufacturing	6.53%	11.40%	37.60%
Other	**-63.12%***	-52.21%	-58.18%
Cancer type			
Occupational	1	1	1
Asbestos	**317.66%*****	**793.34%*****	**458.17%*****
Fire Fighter	57.98%	124.63%	83.02%
Missing	314.29%	432.87%	452.73%
Injury type			
Asbestosis	1	1	
Leukemias	47.15%	89.88%	
Lymphosarcoma and Reticulosarcoma	-59.16%	-25.48%	
Neoplasms and Tumors	-30.71%	9.23%	
Mesothelioma	59.57%	8.20%	
Other	108.65%	**306.86%****	
Unknown	-27.88%	37.37%	
Region			
Halifax, East Shore, West Hants	1	1	
Annapolis Valley, South Shore, South West	77.62%	18.03%	
Colchester-East Hants, Cumberland, Pictou	-65.12%	-21.35%	
Cape Breton, Guysborough, Antigonish	-34.63%	11.94%	
Other	-86.30%	-87.53%	
Missing	**-68.66%*****	-49.12%	
Body part affected			
Abdomen/Digestive	1	1	
Urinary System	94.90%	104.01%	
Body Systems	143.59%	30.30%	
Respiratory System	21.28%	-7.43%	
Circulatory System	-20.07%	-29.38%	
Head and Neck	-68.74%	-70.62%	
Pelvic Region	-46.99%	-30.09%	
Other	-46.34%	-70.97%	
Missing	-25.38%	-65.16%	

Unadjusted and adjusted linear regression models were log transformed. Values shown are exponentiated to estimate the geometric mean, expressed as a percentage of change in total cost compared to the referent

Total cost of claims were adjusted for inflation to 2014 from last year accident claims were reported

Estimates are adjusted for the national (1957-1978) and Nova Scotia (1979-2015) Consumer Price Index for All Items

Inflation was determined using year of biopsy

*** p<0.01, ** p<0.05, * P<0.1

N = 304

Total cost: $37 900 000

Table A3-6 - Determinants of Occupational Cancer Costs in Nova Scotia – Cost Estimate 6

	Unadjusted	Adjusted Full Model	Adjusted Parsimonious Model
Age at biopsy			
<=50	1	1	1
51-64	39.10%	10.55%	9.90%
65+	-53.41%	**-71.27%****	**-70.36%****
Industry			
Government	**1**	**1**	**1**
Construction	186.05%	166.47%	147.37%
Manufacturing	4.11%	12.76%	38.51%
Other	**-63.91%***	-51.82%	-57.95%
Cancer type			
Occupational	1	1	1
Asbestos	**331.10%*****	**812.39%*****	**478.52%*****
Fire Fighter	64.39%	132.94%	91.67%
Missing	318.71%	435.59%	457.56%
Injury type			
Asbestosis	1	1	
Leukemias	50.77%	88.63%	
Lymphosarcoma and Reticulosarcoma	-58.67%	-27.38%	
Neoplasms and Tumors	-31.65%	6.81%	
Mesothelioma	61.49%	6.65%	
Other	99.87%	**294.72%****	
Unknown	-29.46%	32.94%	
Region			
Halifax, East Shore, West Hants	1	1	
Annapolis Valley, South Shore, South West	80.15%	18.09%	
Colchester-East Hants, Cumberland, Pictou	-66.06%	-23.88%	
Cape Breton, Guysborough, Antigonish	-36.49%	10.14%	
Other	-86.65%	-87.66%	
Missing	**-68.29%*****	-48.17%	
Body part affected			
Abdomen/Digestive	1	1	
Urinary System	95.54%	109.01%	
Body Systems	143.56%	29.90%	
Respiratory System	17.45%	-8.64%	
Circulatory System	-19.64%	-29.21%	
Head and Neck	-69.66%	-70.65%	
Pelvic Region	-47.38%	-29.46%	
Other	-46.70%	-71.06%	
Missing	-27.03%	-65.37%	

Unadjusted and adjusted linear regression models were log transformed. Values shown are exponentiated to estimate the geometric mean, expressed as a percentage of change in total cost compared to the referent
Total cost of claims were adjusted for inflation to 2014 from last year accident claims were reported
Estimates are adjusted for the national (1957-1978) and Nova Scotia (1979-2015) Consumer Price Index for Health and Personal Care.
Inflation was determined using year of biopsy
*** p<0.01, ** p<0.05, * P<0.1
N = 304
Total cost: $36 500 000

Table A3-7 - Determinants of Occupational Cancer Costs in Nova Scotia – Cost Estimate 7

	Unadjusted	Adjusted Full Model	Adjusted Parsimonious Model
Age at biopsy			
<=50	1	1	1
51-64	2.94%	-22.49%	-22.07%
65+	**-67.52%***	**-81.86%*****	**-80.00%*****
Industry			
Government	**1**	**1**	**1**
Construction	175.94%	168.8%	160.23%
Manufacturing	2.17%	5.06%	54.57%
Other	**-71.62%***	-62.08%	-65.40%
Cancer type			
Occupational	1	1	1
Asbestos	**344.95%*****	**1292.24%*****	**530.47%*****
Fire Fighter	87.05%	157.10%	130.00%
Missing	530.28%	642.09%	786.05%
Injury type			
Asbestosis	1	1	
Leukemias	156.10%	389.59%	
Lymphosarcoma and Reticulosarcoma	-30.30%	105.88%	
Neoplasms and Tumors	8.30%	91.90%	
Mesothelioma	79.61%	14.49%	
Other	221.65%	**657.54%****	
Unknown	-44.88%	79.91%	
Region			
Halifax, East Shore, West Hants	1	1	
Annapolis Valley, South Shore, South West	111.74%	26.52%	
Colchester-East Hants, Cumberland, Pictou	-58.77%	0.08%	
Cape Breton, Guysborough, Antigonish	-34.10%	19.03%	
Other	-98.63%	**-98.98%****	
Missing	**-74.67%*****	-51.91%	
Body part affected			
Abdomen/Digestive	1	1	
Urinary System	127.32%	121.22%	
Body Systems	185.39%	26.05%	
Respiratory System	12.63%	5.07%	
Circulatory System	-31.63%	-53.42%	
Head and Neck	-72.35%	-74.70%	
Pelvic Region	-37.37%	-4.02%	
Other	-38.34%	-69.38%	
Missing	-63.16%	-81.06%	

Unadjusted and adjusted linear regression models were log transformed. Values shown are exponentiated to estimate the geometric mean, expressed as a percentage of change in total cost compared to the referent

Total cost of claims were adjusted for inflation to 2014 from first year accident claims were reported

Estimates are adjusted for the national (1957-1978) and Nova Scotia (1979-2015) Consumer Price Index for All Items

Inflation was determined using year of biopsy

*** p<0.01, ** p<0.05, * P<0.1

N = 304

Total cost: $44 000 000
